# Polymorphisms in methotrexate transporters and their relationship to plasma methotrexate levels, toxicity of high-dose methotrexate, and outcome of pediatric acute lymphoblastic leukemia

**DOI:** 10.18632/oncotarget.17781

**Published:** 2017-05-10

**Authors:** Shu-Guang Liu, Chao Gao, Rui-Dong Zhang, Xiao-Xi Zhao, Lei Cui, Wei-Jing Li, Zhen-Ping Chen, Zhi-Xia Yue, Yuan-Yuan Zhang, Min-Yuan Wu, Jian-Xiang Wang, Zhi-Gang Li, Hu-Yong Zheng

**Affiliations:** ^1^ Beijing Key Laboratory of Pediatric Hematology Oncology, National Key Discipline of Pediatrics, Ministry of Education, Key Laboratory of Major Diseases in Children, Ministry of Education, Hematology Oncology Center, Beijing Children's Hospital, Capital Medical University, Beijing, 100045, China; ^2^ Institute of Hematology and Blood Diseases Hospital, Chinese Academy of Medical Sciences and Peking Union Medical College, Tianjin, 30020, China

**Keywords:** methotrexate, acute lymphoblastic leukemia, polymorphism, SLCO1B1, SLC19A1

## Abstract

High-dose methotrexate (HDMTX) plays an important role in the treatment of acute lymphoblastic leukemia (ALL) although there is great inter-patient variability in the efficacy and toxicity of MTX. The relationship between polymorphisms in genes encoding MTX transporters and MTX response is controversial. In the present study, 322 Chinese children with standard- and intermediate-risk ALL were genotyped for 12 polymorphisms. *SLCO1B1* rs10841753 showed a significant association with plasma MTX levels at 48 h (*P* = 0.017). Patients who had the *ABCB1* rs1128503 C allele had longer duration of hospitalization than did those with the TT genotype (*P* = 0.006). No association was found between oral mucositis and any polymorphism. Long-term outcome was worse in patients with the *SLCO1B1* rs4149056 CC genotype than in patients with TT or TC (5-year event-free survival [EFS] 33.3 ± 19.2% vs. 90.5 ± 1.7%, *P* < 0.001), and was worse in patients with the *SCL19A1* rs2838958 AA genotype than in patients with AG or GG (5-year EFS 78.5 ± 4.6% vs. 92.2 ± 1.8%, *P* = 0.008). Multiple Cox regression analyses revealed associations of minimal residual disease (MRD) at day 33 (hazard ratio 3.458; *P* = 0.002), MRD at day 78 (hazard ratio 6.330; *P* = 0.001), *SLCO1B1* rs4149056 (hazard ratio 12.242; *P* < 0.001), and *SCL19A1* rs2838958 (hazard ratio 2.324; *P* = 0.019) with EFS. Our findings show that polymorphisms in genes encoding MTX transporters substantially influence the kinetics and response to HDMTX therapy in childhood ALL.

## INTRODUCTION

Methotrexate (MTX) has been widely used as an anticancer agent in most chemotherapy protocols for childhood acute lymphoblastic leukemia (ALL). Despite its clinical success, MTX may cause serve adverse effects and toxicities [1–3]. MTX pharmacokinetics, efficacy, and toxicity have great inter-patient variability. This diversity can be partly explained by sequence differences in genes encoding MTX membrane transporter proteins [4–6].

MTX enters the cell via the solute carrier family 19 member 1 (SLC19A1) or the solute carrier organic anion transporter 1B1 (SLCO1B1) [7, 8]. It is exported from the cells by ATP binding cassette (ABC) transporters, including ABC subfamily B member 1 (ABCB1), and ABC subfamily G member 2 (ABCG2) [4]. Previous studies suggest that variations in single nucleotide polymorphisms (SNPs) of genes encoding MTX transporters play a great role in ALL prognosis and MTX response [9–15], but the results are inconsistent, which might result from small study cohorts, heterogeneous study populations, and differences in treatment protocols.

Therefore, the main goal of the present study was to investigate the influence of SNPs on the pharmacokinetics and toxicity of high-dose MTX (HDMTX), as well as clinical outcome in ALL patients. A total of 12 SNPs in four genes involved in the MTX transport pathway (*SLCO1B1, SLC19A1, ABCB1*, and *ABCG2*) were selected. The association study was carried out in 322 Chinese children with ALL treated according to the Beijing Children's Hospital (BCH)-2003 and Chinese Childhood Leukemia Group (CCLG)-2008 protocols.

## RESULTS

### Patient and genotyping characteristics

Patient characteristics are shown in Supplementary Table 1. No significant differences were observed between patients included (*n* = 322) and those not included (*n* = 142) in this study.

We genotyped 12 SNPs in four genes (*SLCO1B1, SLC19A1, ABCB1*, and *ABCG2*). The selected SNPs and allele frequencies are shown in Supplementary Table 2 with an average call rate of 98.23%. *ABCB1* rs2032582 was excluded from the following analyses because its allele frequencies deviated from HWE.

### SNPs with pharmacokinetics

To explore the impact of SNPs on MTX pharmacokinetics, we tested the association between selected SNPs and MTX plasma level at 48 h from the first dose of HDMTX infusion. Plasma MTX data were available for 317 patients with the range from 0.09 μmol/L to 41.63 μmol/L (median: 0.45 μmol/L). We found a statistically significant association between plasma MTX level and the *SLCO1B1* rs10841753. Patients with CC genotypes had a lower MTX plasma level than did patients with the T allele (CT+TT) (*P* = 0.017) (Table 1 and Supplementary Figure 2A). The MTX plasma concentration at 48 h was higher in patients with the *SLCO1B1* rs4149056 CC genotype compared with those with the T allele (TC+TT) (1.1μmol/L vs. 0.45 μmol/L, *P* = 0.441), and was lower in patients with the *SLCO1B1* rs11045879 TT genotype compared with those with the C allele (CC+TC) (0.39 μmol/L vs.0.50 μmol/L, *P* = 0.287). The trends were consistent with previous studies, but the association did not reach statistical significance (Table 2 and Supplementary Figure 2B and 2C). Both of the patients who died after receiving the first course of HDMTX had a high plasma MTX level at 48 h (13.09 μmol/L and 8.93 μmol/L, respectively). Their genotypes for *SLCO1B1* rs10841753, rs4149056, rs11045879, and rs2306283 were TC, TT, TC, TC, and TT, TC, TC, CC, respectively. We also analyzed the association between polymorphisms and MTX plasma levels during administration of the four courses by repeated measures ANOVA; however, none of the associations was significant.

**Table 1:** BCH-2003 and CCLG-2008 treatment protocol

	Stratification Criteria	Treatment Block						
		Remission induction	Early intensification	Consolidation	Delay Intensification I	Maintenance I	Delay intensification II	Maintenance II
BCH-2003								
SR	1 ≤ age < 6 WBC < 20 × 10^9^/L Good prednisone response No T-cell and not mature B-cell No t(9;22) or MLL rearrangements Bone marrow morphology was M1 at day 33	VDLP (DNR×2)	CAM	HDMTX 3g/m^2^×4	VDLD+CAM	6-MP+MTX/VD+IT	HDMTX	6-MP+MTX/VD+IT
IR	Good prednisone response No t(9;22) Bone marrow morphology was M1 at day 33 Any one of : Age ≥ 6 or < 1 WBC ≥ 20 × 10^9^/L T-cell MLL rearrangement	VDLP (DNR×4)	CAM×2	HDMTX 5g/m^2^×4	VDLD+CAM	6-MP+MTX/VD+IT	VDLA+VM26+ HD-Ara-c	6-MP+MTX/VD+IT
CCLG-2008	1 ≤ age < 10 WBC < 50 × 10^9^/L Good prednisone response No T-cell and not mature B-cell							
SR	No t(9;22), t(1;19) or MLL rearrangements Bone marrow morphology was M1/M2 at day 15 and M1 at day33 No CNSL MRD < 10^–4^ at day 33	VDLP (DNR×2)	CAM	HDMTX 2g/m^2^×4	VDLD+CAM	/	/	6-MP+MTX/VD+IT
IR	Good prednisone response No t(9;22) or MLL rearrangements Bone marrow morphology at day 15 was M1/M2 with IR protocol or M3 with SR protocol MRD < 10^–2^ at day 33 and < 10^–3^ at day 78 Any one of : Age ≥ 10 or < 1 WBC ≥ 50 × 10^9^/L T-cell CNSL with no other high risk factor	VDLP (DNR×4)	CAM×2	HDMTX 5g/m^2^×4	VDLD+CAM	6-MP+MTX	VDLD+CAM	6-MP+MTX/VD+IT

WBC: white blood cell; SR: standard risk; IR: Intermediate-risk. MRD: minimal residual disease. CNSL: central nervous system leukemia.

**Table 2:** Association of selected SNPs with plasma levels of MTX at 48 h from the first MTX infusion

SNPs	Median (Min-Max) (μmol/L)	*P*
*SLCO1B1* rs11045879		0.287
CC +TC	0.50 (0.09–41.63)	
TT	0.39 (0.12–19.88)	
*SLCO1B1* rs4149056		0.441
TC+TT	0.45 (0.09–41.63)	
CC	1.1. (0.15–7.53)	
*SLCO1B1* rs2306283		0.294
CC +TC	0.45 (0.09–41.63)	
TT	0.37 (0.12–3.91)	
*SLCO1B1* rs10841753		0.017
TC+TT	0.47 (0.09–41.63)	
CC	0.32 (0.14–5.50)	
*SLC19A1* rs1051266		0.384
AA +AG	0.41 (0.09–34.05)	
GG	0.59 (0.14–41.63)	
*SLC19A1* rs3788200		0.491
AG+GG	0.45 (0.09–41.63)	
AA	0.47 (0.11–34.05)	
*SLC19A1* rs1131596		0.219
TC+CC	0.41 (0.09–34.05)	
TT	0.6 (0.14–41.63)	
*SLC19A1* rs2838958		0.259
AG+GG	0.41 (0.09–34.05)	
AA	0.59 (0.16–41.63)	
*ABCB1* rs1128503		0.149
TC+CC	0.47 (0.09–19.88)	
TT	0.39 (0.12–41.63)	
*ABCB1* rs1045642		0.340
TC+TT	0.41 (0.12–41.63)	
CC	0.46 (0.09–10.88)	
*ABCG2* rs2231137		0.688
AG+GG	0.45 (0.09–41.63)	
AA	0.46 (0.15–10.29)	

SNP: single nucleotide polymorphism.

### SNPs with MTX-induced toxicity and duration of hospitalization for HDMTX treatment

Oral mucositis is a common side effect attributed to the use of HDMTX. To investigate genetic determinants for oral mucositis after HDMTX, information regarding oral mucositis during the consolidation therapy period was available for 317 patients. Table 3 shows the risk evaluation for the development of grade 3–4 oral mucositis, calculated by univariate analysis. A total of 38 patients developed grade 3–4 oral mucositis (12.0%). No polymorphism was found to be associated with oral mucositis, although it occurred more frequently in patients with the *ABCB1* rs1128503C allele (CC and TC) than in those with the TT genotype (OR 2.009, 95% CI 0.987–4.090, *P* = 0.054). With regard to *SLCO1B1* rs4149056, patients with the C allele had a lower risk of developing mucositis. None of the six patients with the CC genotypes developed oral mucositis. The frequency was 8.47% for patients with TC, and 13.25% for those with TT; however, the differences among genotypes were not statistically significant (Table 3 and Supplementary Figure 3).

**Table 3:** Logistic regression analysis of association between selected SNPs and oral mucositis (grade 3–4)

SNP (*n*)	Oral mucositis *n* (%)	OR (95% CI)	*P*
*SLCO1B1* rs11045879			
CC (52)	6 (11.54)	Reference	
TC (136)	18 (13.24)	1.177 (0.558–2.480)	0.669
TT (122)	14 (11.46)	1.006 (0.364–2.781)	0.990
TC+TT vs. CC		0.921 (0.364–2.330)	0.862
*SLCO1B1* rs4149056			
CC (6)	0	Reference	
TC (59)	5 (8.47)	0.606 (0.226–1.626)	0.320
TT (249)	33 (13.25)	0	0.999
TC+TT vs. CC		0	0.999
*SLCO1B1* rs2306283			
CC (170)	23 (13.53)	Reference	
TC (115)	13 (11.30)	1.643 (0.361–7.478)	0.521
TT (23)	2 (8.70)	1.338 (0.281–6.375)	0.715
TC+TT vs. CC		1.283 (0.641–2.566)	0.481
*SLCO1B1* rs10841753			
CC (33)	4 (12.12)	Reference	
T C (120)	17 (14.17)	1.379 (0.672–2.828)	0.381
TT (159)	17 (10.69)	1.152 (0.361–3.676)	0.811
TC+TT vs. CC		0.994 (0.329–3.001)	0.991
*SLC19A1* rs1051266			
AA (73)	7 (9.59)	Reference	
A G(142)	20 (14.08)	1.252 (0.570–2.749)	0.576
GG (95)	11 (11.58)	0.810 (0.298–2.204)	0.680
AG+GG vs. AA		0.705 (0.297–1.675)	0.428
*SLC19A1* rs3788200			
AA (73)	7 (9.59)	Reference	
A G(143)	20 (13.99)	1.271 (0.579–2.789)	0.549
GG (97)	11 (11.34)	0.829 (0.305–2.255)	0.714
AG+GG vs. AA		0.715 (0.301–1.699)	0.448
*SLC19A1* rs1131596			
TT (101)	11 (10.89)	Reference	
T C(144)	20 (13.89)	1.117 (0.411–3.039)	0.828
CC (71)	7 (9.86)	1.475 (0.592–3.671)	0.404
TC+CC vs. TT		0.851 (0.404–1.792)	0.671
*SLC19A1* rs2838958			
AA (89)	10 (11.24)	Reference	
AG (145)	19 (13.10)	0.970 (0.373–2.526)	0.951
GG (78)	9 (11.54)	1.156 (0.496–2.693)	0.737
AG+GG vs. AA		0.882 (0.409–1.900)	0.748
*ABCB1* rs1128503			
TT (153)	13 (8.50)	Reference	
T C(121)	18 (14.88)	1.882 (0.882–4.014)	0.102
CC (38)	7 (18.42)	2.432 (0.897–6.596)	0.081
TC+CC vs. TT		2.009 (0.987–4.090)	0.054
*ABCB1* rs1045642			
CC (118)	14 (11.86)	Reference	
T C(148)	18 (12.16)	0.965 (0.348–2.676)	0.945
TT (49)	6 (12.24)	0.992 (0.370–2.660)	0.988
TC+TT vs. CC		0.970 (0.481–1.959)	0.933
*ABCG2* rs2231137			
AA (30)	5 (16.67)	Reference	
AG (123)	13 (10.57)	0.821 (0.391–1.724)	0.603
GG (159)	20 (12.58)	1.390 (0.478–4.406)	0.546
AG+GG vs. AA		1.509 (0.54104.212)	0.432

SNP: single nucleotide polymorphism; OR: odds ratio; CI: confidential interval.

To study the impact of SNPs on duration of hospitalization for HDMTX treatment, we analyzed the association between genotypes and average length of hospitalization for the four courses of HDMTX treatment. Table 4 shows that patients with the *ABCB1* rs1128503 C allele had prolonged duration of hospitalization compared with those with the TT genotype (mean 5.92 vs. 5.51 days, *P* = 0.006).

**Table 4:** Association of selected SNPs with duration of hospitalization for HDMTX treatment

SNPs	Mean ± SD (days)	*P*
*SLCO1B1* rs11045879		0.355
CC +TC	5.77 ± 1.39	
TT	5.62 ± 1.21	
*SLCO1B1* rs4149056		0.634
TC+TT	5.72 ± 1.33	
CC	5.46 ± 0.62	
*SLCO1B1* rs2306283		0.345
CC +TC	5.73 ± 1.35	
TT	5.46 ± 1.04	
*SLCO1B1* rs10841753		0.988
TC+TT	5.72 ± 1.34	
CC	5.72 ± 1.35	
*SLC19A1* rs1051266		0.544
AA +AG	5.69 ± 1.20	
GG	5.79 ± 1.59	
*SLC19A1* rs3788200		0.339
AG+GG	5.75 ± 1.44	
AA	5.58 ± 0.85	
*SLC19A1* rs1131596		0.522
TC+CC	5.68 ± 1.20	
TT	5.79 ± 1.58	
*SLC19A1* rs2838958		0. 906
AG+GG	5.70 ± 1.19	
AA	5.72 ± 1.62	
*ABCB1* rs1128503		0.006
TC+CC	5.92 ± 1.37	
TT	5.51 ± 1.28	
*ABCB1* rs1045642		0.112
TC+TT	5.63 ± 1.33	
CC	5.88 ± 1.34	
*ABCG2* rs2231137		0.094
AG+GG	5.67 ± 1.29	
AA	6.18 ± 1.59	

SNP: single nucleotide polymorphism.

### Haplotypes with pharmacokinetics and MTX-induced toxicity

To test the association between haplotypes and MTX plasma levels/toxicity, we first determined the linkage disequilibrium (LD) block structure for each gene. Supplementary Figure 4 shows that *SLCO1B1* rs10841753, rs2306283, rs4149056, and *SLC19A1* rs3788200, rs1051266, rs1131596 were in LD, respectively. None of the haplotypes was associated with MTX level or MTX-induced oral mucositis (Supplementary Tables 3 and 4).

### SNPs with treatment outcome

**Figure 1:**
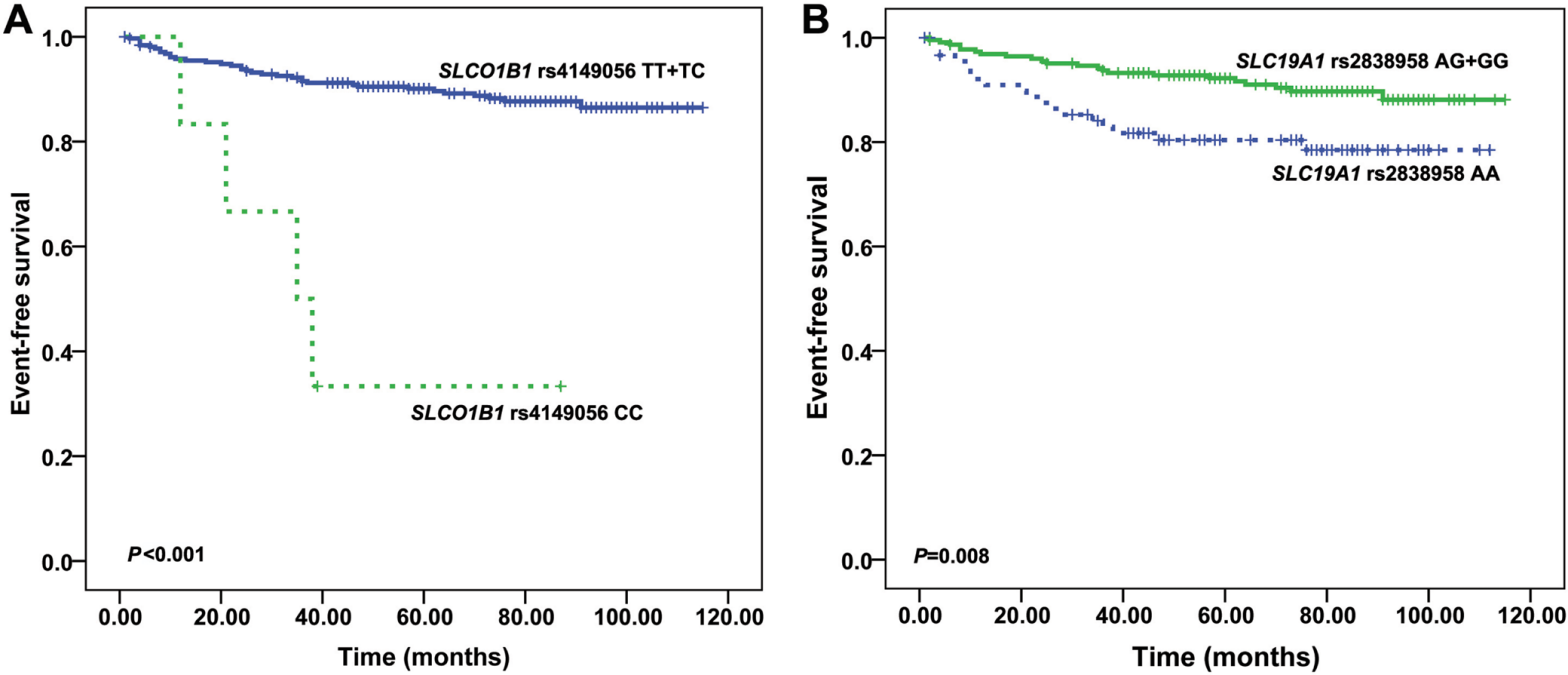
Polymorphisms and event-free survival (EFS) (**A**) The Kaplan-Meier survival curve is shown to illustrate the difference between *SLCO1B1* rs4149056 CC variant and TT/TC variants (log-rank *P* < 0.001). (**B**) The Kaplan-Meier survival curve is shown to illustrate the difference between *SLC19A1* rs2838958 AA variant and GG/AG variants (log-rank *P* = 0.008).

To define the influence of selected SNPs on treatment outcomes of ALL patients, we did both univariate and multivariate analyses. Univariate analyses showed that *SLCO1B1* rs4149056 and *SLC19A1* rs2838958 were significantly associated with the risk of events (*P* < 0.001 and 0.008, respectively). Compared with patients with the rs4149056 T allele (TC+TT), patients with the CC genotype had a poorer outcome (5-year EFS 33.3 ± 19.2% vs. 90.5 ± 1.7; Figure 1A). Among the six patients with the rs4149056 CC genotype, three patients relapsed and one died of infection. The *SLC19A1* rs2838958 AA genotype was associated with poorer outcome than was the G allele (AG+GG) (5-year EFS 78.5 ± 4.6% vs 92.2 ± 1.8%; Figure 1B). In our study, the *SLC19A1* rs1051266 A allele (AA+AG) was associated with better outcome than was the GG genotype (5-year EFS 91.9 ± 1.9% vs. 82.1 ± 4.1%; Supplementary Figure 5), but this association was not statistically significant (*P* = 0.08).

In a multivariate Cox regression analysis that included immunophenotype, WBC, *TEL/AML1*, MRD class (day 33 and day 78), and *SLCO1B1* rs4149056 and *SLC19A1* rs2838958 genotypes as independent variables, *SLCO1B1* rs4149056 and *SLC19A1* rs2838958 genotypes and MRD class were shown to be independent prognostic factors (Table 5). The adjusted hazard ratios were 12.242 (95% CI = 3.937–38.059; *P* < 0.001) for patients with the *SLCO1B1* rs4149056 CC genotype vs those with the T allele (TC+TT). The *SLC19A1* rs2838958 AA genotype still had a poorer prognosis than did the G allele (AG+GG) (hazard ratio, 2.324, 95% CI 1.147–4.710; *P* = 0.019). In addition, the presence of MRD at day 33 and day 78 also had significant effect on outcome (hazard ratio, 3.458, 95% CI 1.598–7.483; *P* = 0.002; hazard ratio, 6.330, 95% CI 2.181–18.369; *P* = 0.001). Other clinical features, such as WBC, *TEL-AML1*, and immunophenotype were not independent prognostic factors.

**Table 5:** Multivariate Cox regression analysis for EFS

	HR	95% CI	*P*
Immunophenotype			
B-ALL	1	Reference	0.870
T-ALL	1.094	0.371–3.230	
WBC			
< 50 × 10^9^	1	Reference	0.059
≥ 50 × 10^9^	2.385	0.968–5.877	
TEL-AML1			
Negative	1	Reference	0.935
Positive	0.959	0.344–2.668	
MRD at day 33			
< 10^–4^	1	Reference	0.002
≥ 10^–4^	3.458	1.598–7.483	
MRD at day 78			
< 10^–3^	1	Reference	0.001
≥ 10^–3^	6.330	2.181–18.369	
*SLCO1B1* rs4149056			
TT+TC	1	Reference	< 0.001
CC	12.242	3.937–38.059	
*SLC19A1* rs2838958			
AG+GG	1	Reference	0.019
AA	2.324	1.147–4.710	

EFS: event-free survival; WBC: white blood cell; MRD: minimal residual disease; HR: hazard ratio; CI: confidence interval.

## DISCUSSION

HDMTX plays an important role in the improvement of cure rates in ALL. There is great inter-individual variability in MTX response [4, 5, 16, 17]. Several SNPs in genes involved in the MTX transporter pathway have been implicated in the kinetics and effects of MTX in previous studies [9–15], but some results are ambiguous. In the present study, we evaluated the correlation of 12 polymorphisms in four genes involved in the MTX transport pathway with pharmacokinetics and toxicity of HDMTX as well as outcomes in 322 Chinese ALL patients.

*SLCO1B1* rs10841753 showed a significant association with the MTX plasma level at 48 h from the first dose of HDMTX infusion in our study. Lower plasma MTX concentrations were found in patients with the rs10841753 CC genotype than in patients with TC or TT genotype. The *SLCO1B1* gene encodes an organic anion transporter (OATP1B1) that is located on the sinusoidal membrane of human hepatocytes, and mediates disposition of many medications [18–20]. Previous studies reported that SLCO1B1 can transport MTX *in vitro* [21, 22], and *in vivo* [23]. Although many SNPs have been identified in the *SLCO1B1* gene, only a few are known to have functional significance [24]. The common *SLCO1B1* rs4149056 variant results in decreased transport *in vitro* [8], whereas the *SLCO1B1* rs2306283 variant increases the transport activity for MTX *in vitro* [22]. The genome-wide study by Trevino et al. demonstrated that *SLCO1B1* rs11045879, rs4149056, and eight additional SNPs including rs10841753 are strongly associated with MTX clearance in childhood ALL [25]. The functional rs2306283 in *SLCO1B1* was recognized by Ramsey et al. as a predictor of MTX clearance in ALL patients [22]. The association between *SLCO1B1* rs4149056 and MTX clearance was confirmed in subsequent studies [5, 14, 15, 26]. Li et al. replicated the association between *SLCO1B1* rs11045879 and MTX plasma levels in 280 Chinese pediatric B–ALL patients [27]; however, other studies show conflicting results. Lopez-Lopez et al. failed to show a statistically significant association between rs11045879 and MTX plasma concentrations in patients with ALL (corrected *P* = 0.08) [12]. In our study, we could not validate the influence of *SLCO1B1* rs4149056, rs11045879, and rs2306283 on MTX pharmacokinetics. Our results showed that lower MTX plasma concentration at 48 h was found in patients with the rs4149056 T allele (TC+TT) than in patients with the CC genotype (0.45 μmol/L vs. 1.1 μmol/L), in patients with the rs11045879 TT genotype compared with the CC or TC genotype (0.39μmol/L vs.0.50μmol/L), and in patients with the rs2306283 TT genotype compared with the CC or TC genotype (0.37 μmol/L vs. 0.45 μmol/L), which was consistent with previous results. However, the association did not reach statistical significance (*P* = 0.441, 0.287, 0.294, respectively). Trevino et al. reported that *SLCO1B1* genetic variation accounted for only 9.3% of the MTX inter-patient variability, compared with 17.9% for the treatment regimen [25]. Thus, the differences between our protocol and other protocols, including MTX dosage, the methodology of leucovorin rescue, and concomitant hydration and alkalinization may be the possible reasons for the inconsistent results. *SLCO1B1* rs10841753 is an SNP that is located in the intron of the gene. It is unknown how this variant affects the function of *SLCO1B1*, perhaps by effects on transcription and post-transcriptional processing [25]. We replicated the association between MTX plasma level with rs10841753, confirming that *SLCO1B1* was a predictor for MTX pharmacokinetics in our cohort, and that different polymorphisms can affect the functions of *SLCO1B1* and MTX clearance.

Because altered MTX pharmacokinetics may affect adverse drug reactions and treatment efficacy, MTX toxicity and survival were also investigated in this study. Oral mucositis is a common side effect attributed to the use of HDMTX. One previous study found that *SLCO1B1* rs4149056 was associated with the risk of mucositis [15]; however, this association was not replicated in two other studies [14, 25]. In our study, patients with the *SLCO1B1* rs4149056 C allele had a lower risk of developing mucositis. None of the six patients with the CC genotype developed oral mucositis, but the differences among genotypes were not statistically significant. Several factors, including differences in early symptomatic treatment for toxicity, toxicity criteria, treatment protocols, and sample sizes of groups may be responsible for the inconsistent results. Compared with patients with the TT genotype, those with the *ABCB1* rs1128503C allele had a higher risk of developing oral mucositis after receiving HDMTX (*P* = 0.054)), and had a longer duration of hospitalization for the HDMTX treatment (*P* = 0.006). Zgheib et al. found that rs1128503 was associated with neutropenia [28]. It is possible that the *ABCB1* rs1128503C allele is associated with several different manifestations of drug toxicity, which may lead to prolongation of the treatment period. A larger study should be performed to confirm the association.

We next analyzed the prognostic roles of selected polymorphisms in childhood ALL. Our results showed that *SLCO1B1* rs4149056 and *SLC19A1* rs2838958 were independent prognostic factors in our study. Zhang et al. found that rs4149056 was not associated with the risk of relapse in 136 Chinese pediatric ALL patients. But interestingly, their results showed that patients with the C allele (CC+TC) were given higher leucovorin doses than were patients with the TT genotype [15]. In addition, Skaby et al. reported that higher leucovorin doses during HDMTX treatment may raise the risk for relapse [29]. Based on these data, it seemed that the rs4149056 C allele should be associated with higher risk for relapse. We found that patients with the rs4149056 CC genotype had poorer outcome than did those with the TC or TT genotype (5-year EFS 33.3 ± 19.2%% vs. 90.5 ± 1.7; Figure 1A), which were theoretically reasonable. The sample size in the study by Zhang et al. was relatively small; there were only two patients with the rs4149056 CC alleles, which might obscure the prognostic role of rs4149056. Furthermore, the median follow-up time in the present study was longer than the follow-up time in the study by Zhang et al (75 months vs. 60 months), which is important because relapses tend to occur late in many low-risk ALL patients [15]. Thus, the correlation between rs4149056 and EFS should be further validated.

It is noteworthy that we also found that *SLC19A1* rs2838958 was an independent prognostic factor in our study. *SLC19A1* encodes the reduced folate carrier (RFC) which delivers folate into cells [10]. *SLC19A1* rs2838958 is a tag SNP of *SLC19A1* in the CHB population and is located in the intron of the gene. Univariate analysis showed that *SLC19A1* rs2838958 was significantly associated with the risk of adverse events (*P* = 0.008). The *SLC19A1* rs2838958 AA genotype was associated with poorer outcome than was the G allele (AG+GG) (5-year EFS 78.5 ± 4.6% vs 92.2 ± 1.8%; Figure 1B). In a multivariate Cox regression analysis, the *SLC19A1* rs2838958 AA genotype still was associated with a poorer prognosis than was the G allele (AG+GG) (hazard ratio, 2.324, 95% CI 1.147–4.710; *P* = 0.019). As far as we know, this is the first study to correlate this polymorphism with outcome in pediatric ALL; however, the exact mechanism of rs2838958 is unknown. The rs1051266 (80G>A, His27Arg) was the most studied SNP in the gene *SLC19A1*. In previous studies, there were conflicting results on the prognostic significance of *SLC19A1* rs1051266 [9, 10, 14, 30]. Several studies have previously investigated the effect of rs1051266 on outcome in childhood ALL, but the results were inconsistent. Rocha et al. and Radtke et al. could not demonstrate any significant effect on cure rates in 246 and 499 children with ALL, respectively [14, 30], although Laverdiere et al., in a study of 204 patients, found a reduced rate of relapse for patients with the G-allele, and Gregers et al., in a study of 500 patients, found a decreased EFS rate in patients who had the G-allele [9, 10]. Our data showed that patients with the *SLC19A1* rs1051266 GG genotype had a poorer outcome than did patients with AA or AG genotypes (5-year EFS 82.1 ± 4.1%; vs. 91.9 ± 1.9%, Supplementary Figure 5), but this association was not statistically significant (*P* = 0.08).

This study has some limitations. It is a retrospective study with a modest sample size and limited SNPs in the MTX transport pathway. In addition, oral mucositis was the only manifestation of MTX toxicity that we studied; we did not include other common adverse drug reactions. Further study and replication in an independent cohort are needed to confirm our recent results.

In conclusion, our results show that polymorphisms in the MTX transport pathway are associated with MTX plasma levels and outcome in childhood ALL, which may help to guide and optimize MTX treatment in patients with childhood ALL.

## MATERIALS AND METHODS

### Patients and treatment

Between October 1, 2006 and August 1, 2010, 553 Chinese children were newly diagnosed with acute lymphoblastic leukemia in Beijing Children's Hospital. Of these, patients with standard-risk ALL (SR) and intermediate-risk (IR) were included in this study if a sufficient sample of good quality DNA was available. A total of 322 patients (aged 1.0–15.0 years; median: 4.0 years) were eligible according to these criteria (Supplementary Figure 1). The BCH-2003 protocol was followed in 104 patients between October 2006 and March 2008, and the CCLG-2008 protocol was followed in 218 patients after April 2008. Of these, 117 patients were classified as having SR ALL, and 205 as having IR ALL. Details of the stratification and treatment regimens of the two protocols have been published previously [16] and are outlined in Table 1. At a median follow-up time of 75 months (range, 1.0–115.0), 278 patients were in first remission, 33 patients had relapsed, five had died of severe infection (one patient before the first HDMTX), two had died of severe HDMTX toxicity, and four patients had been lost follow-up before the first HDMTX. The BCH-2003 and CCLG-2008 protocols were approved by the Beijing Children's Hospital Institutional Ethics Committee. Informed consent was obtained from all guardians.

### Minimal residual disease (MRD) analysis

MRD was measured using RQ-PCR-based quantification of IgH gene rearrangements, as previously reported [31–33]. In this study, 297 samples were examined for MRD at day 33, and 278 samples were examined at day 78. Patients with an MRD <10^–4^ at day 33 were considered to have a good response to early treatment. An MRD> 10^–2^ at day 33 or an MRD >10^–3^ at day 78 represented a poor response.

### Candidate gene and polymorphism selection

The selection of candidate genes and SNPs was based on the results of genome-wide association studies, candidate gene association studies, and tag SNPs for CHB (Han Chinese in Beijing) population selected from the HapMap database (http://hapmap.ncbi.nlm.nih.gov/). A total of 12 SNPs in four genes encoding MTX membrane transporter proteins were selected. Detailed information regarding the selected genes and SNPs is shown in Supplementary Table 1. The SNP inclusion criteria were minor allele frequency (MAF)>10%, and in that the gene was demonstrably in Hardy-Weinberg equilibrium (HWE) in the population.

### DNA extraction and genotyping

Genomic DNA was extracted using a Genomic DNA Isolation Kit (U-gene, Anhui, China) from bone marrow specimens at diagnosis according to the manufacturer's instructions. Genotyping was performed on the Sequenom MassARRAY (San Diego, CA, USA) platform in OEbiotech Corporation in Shanghai, China. SNPs with genotyping call rate less than 95% were excluded.

### HDMTX

Patients received four courses of MTX every 2 weeks during the consolidation phase of chemotherapy (day 8, 22, 36, 50). MTX was administered as follows: 10% of the total dose was administered by intravenous infusion over 0.5 hours, and the remaining 90% over 23.5 hours (MTX doses for each treatment arms are shown in Table 1). Leucovorin rescue was initiated with a 15 mg/m^2^ dose at 42 h after the start of the HDMTX infusion, and given twice more, at 48 and 54 h after the infusion. A high plasma MTX level after 48 h (> 0.25 μmol/L) was defined as an indication for prolonged rescue. Monitoring of MTX concentration in plasma was carried out every day until the level was below 0.2 umol/L.

Blood samples of 2 ml were collected at 48 h after the continuous MTX infusion over 24 h. The MTX plasma levels were evaluated by fluorescence polarization immunoassay according to the manufacturer's instructions (TDx Abbott Laboratories, Chicago, IL). Before receiving HDMTX therapy, one patient died from infection and four patients were lost to follow-up. Thus, plasma MTX data at 48 h was available for 317 patients. After receiving the first course of HDMTX, one patient died from Stevens-Johnson syndrome and one from severe gastrointestinal mucositis and infection.

### Toxicity

Oral mucositis is a common manifestation of toxicity that has been attributed specifically to HDMTX [14]. In this study, oral mucositis was graded according to the National Cancer Institute (NCI) Common Terminology Criteria for Adverse Events version 4.0 scoring system (http://evs.nci.nih.gov/ftp1/CTCAE/CTCAE_4.03_2010-06-14_QuickReference_5x7.pdf). The most severe mucositis (Grade 3–4) observed in each patient during the consolidation therapy period was recorded objectively from electronic patient files (blinded to genotypes and MTX levels).

### Statistical analysis

The allele frequencies of all polymorphisms were tested for HWE using χ^2^. An SNP was excluded from the statistical analysis if the frequencies of the wild type and variant alleles deviated from the HWE requirement. The association between MTX concentrations (first course of HDMTX) and polymorphisms was evaluated by the Mann-Whitney *U* test. The association between MTX concentrations of the four courses of HDMTX and polymorphisms was evaluated by repeated measures ANOVA. The differences in duration of hospitalization between genotypes were compared using Student's *t* test. Toxicity was represented by the value 1 or 0, indicating whether an adverse event did or did not occur during the MTX course. Statistical associations between the susceptibility to toxicity and the polymorphisms were analyzed using logistic regression. Haploview v.4.2 was used to determine the haplotype block and to infer haplotype frequencies between individuals with and without toxicity. The duration of event-free survival (EFS) was defined as the time from diagnosis to the date of relapse or death for any reasons (whichever came first), or the last contact with patients in continuous hematologic remission. EFS probabilities were estimated using the Kaplan-Meier method. Differences between groups were compared using the log-rank test. Multivariate Cox regression models adjust for potential confounding variables were used to analyze the prognostic significance of candidate polymorphisms. Two-sided *P*-values < 0.05 were considered statistically significant. SPSS 16.0 software (SPSS Inc, Chicago, IL, USA) was used for statistical analysis.

## SUPPLEMENTARY MATERIALS FIGURES AND TABLES


