# Supplementary material for: Polymorphisms in methotrexate transporters and their relationship to plasma methotrexate levels, toxicity of high-dose methotrexate, and outcome of pediatric acute lymphoblastic leukemia
Source: Oncotarget. 2017 May 10;8(23):37761–72. doi: 10.18632/oncotarget.17781 (PMC5514947; doi:10.18632/oncotarget.17781)
Supplement: Supplementary file 1 [file oncotarget-08-37761-s001.pdf]

Polymorphisms in methotrexate transporters and their relationship to plasma methotrexate levels, toxicity of high-dose methotrexate, and outcome of pediatric acute lymphoblastic leukemia

Supplementary Materials

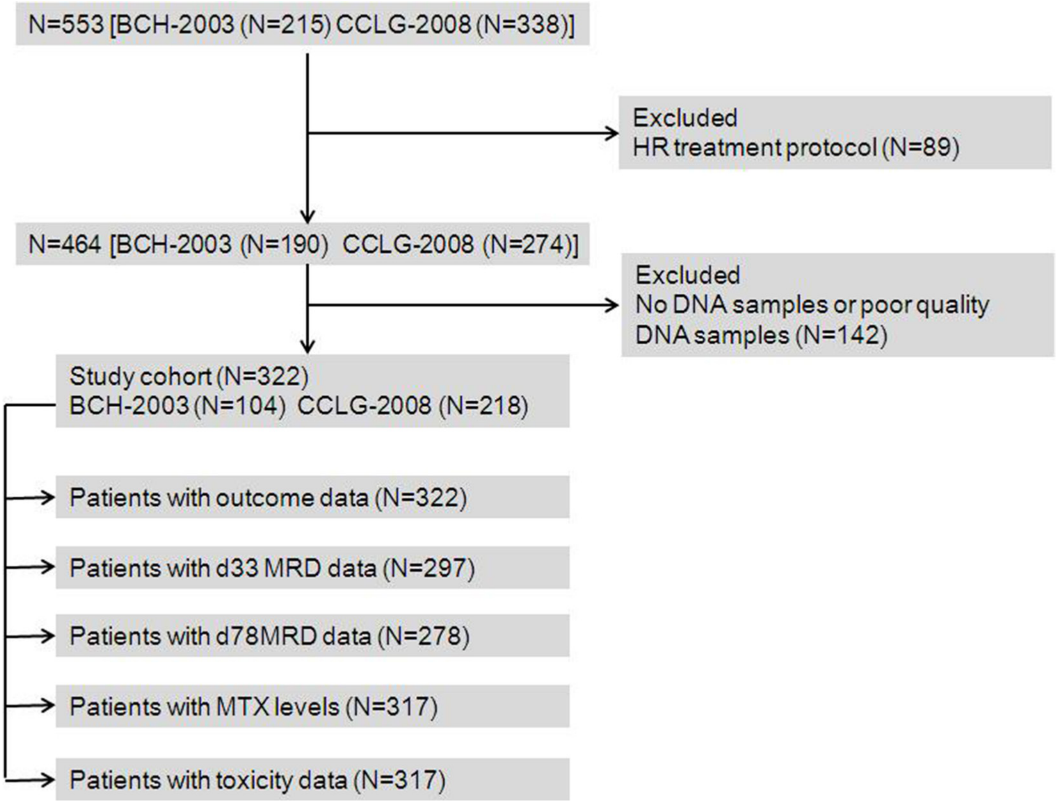

Supplementary Figure 1: Flow chart of patients included in this analysis.

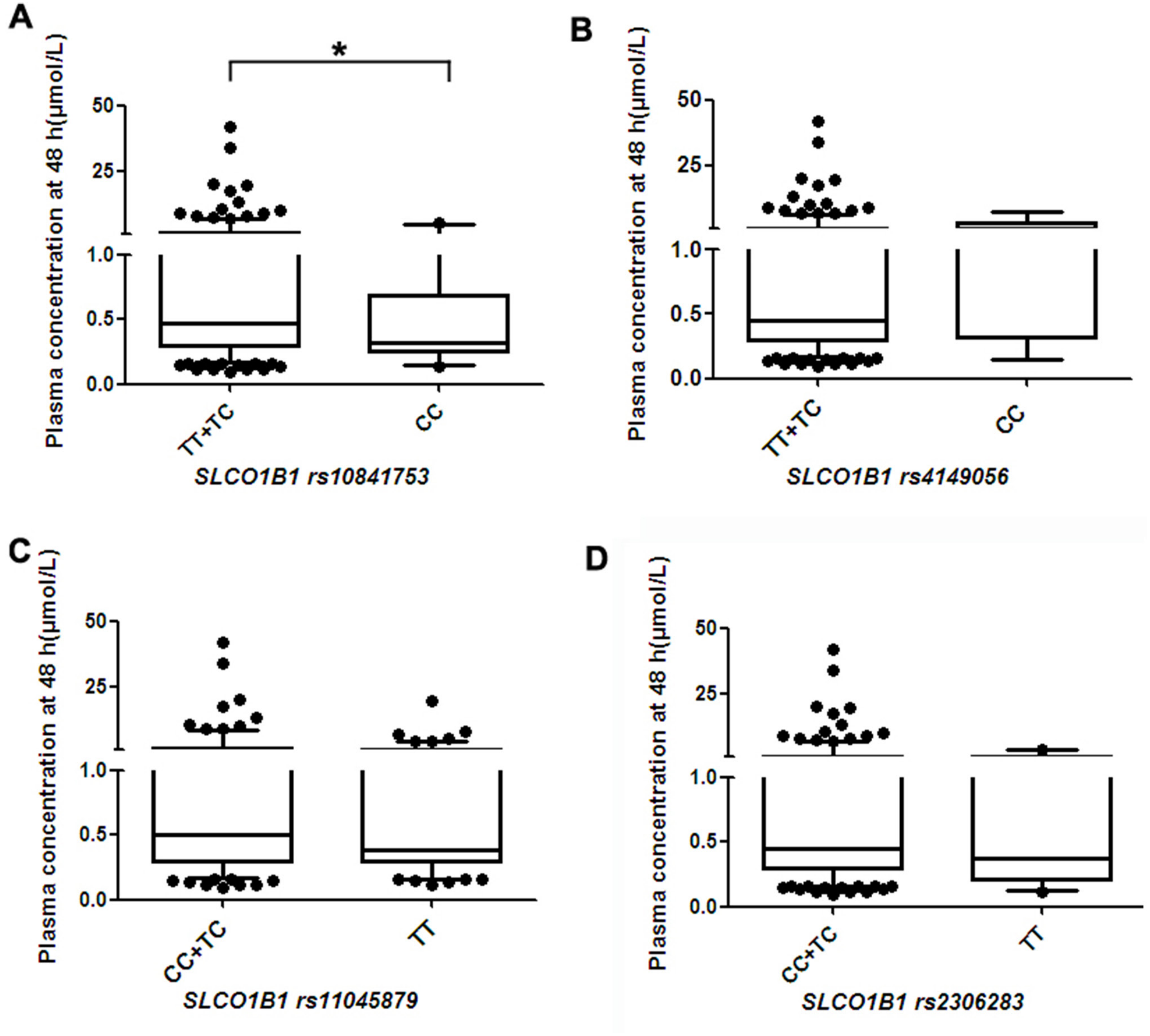

Supplementary Figure 2: *SLCO1B1* polymorphisms and MTX plasma concentration at 48 h. Median is indicated by horizontal line. Boxes indicate 5th–95th percentile. The black dots indicate outliers. * indicates $P < 0.05$. (**A**) *SLCO1B1* rs10841753 variants and plasma MTX levels. (**B**) *SLCO1B1* rs4149056 variants and plasma MTX levels. (**C**) *SLCO1B1* rs111045879 variants and plasma MTX levels. (**D**) *SLCO1B1* rs2306283 variants and plasma MTX levels.

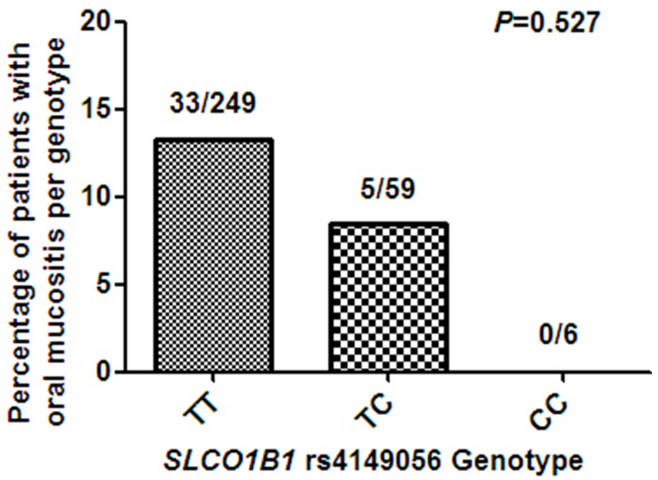

Supplementary Figure 3: *SLCO1B1* rs4149056 and the occurrence of oral mucositis during consolidation therapy. The bar graphs display the percentage of patients who suffered oral mucositis. Numbers above each bar represent the numbers of patients who suffered mucositis vs. the total number of patients for the specific genotype.

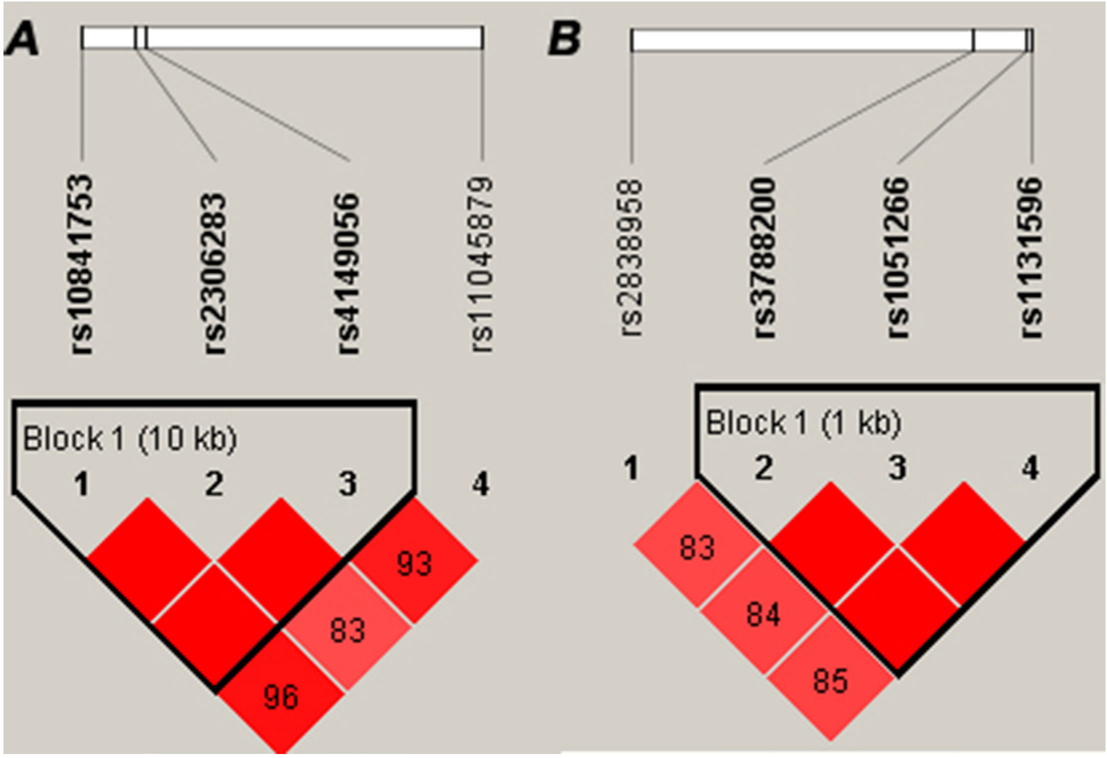

Supplementary Figure 4: Gene map and linkage disequilibrium plot of *SLCO1B1* (**A**) and *SLC19A1* (**B**). Numbers in squares are *D*-values.

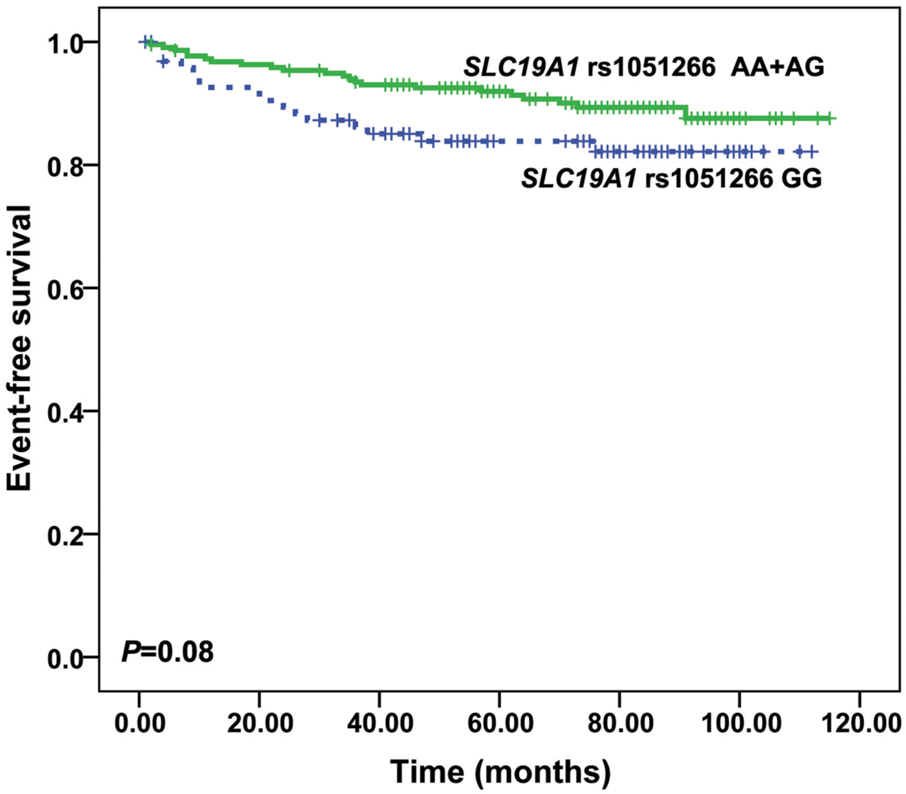

Supplementary Figure 5: *SLC19A1* rs1051266 polymorphism and event-free survival (EFS). The Kaplan-Meier survival curve is shown to visualize the difference between the *SLC19A1* rs1051266 GG variant and AA/AG variants (log-rank $P = 0.08$).

Supplementary Table 1: Patient characteristics

Patient characteristics	BCH-2003 and CCLG-2008 participants with SR and IR risk analyzed	BCH-2003 and CCLG-2008 participants with SR and IR risk not analyzed	*P*
Total number of patients	322	142	
Age			
< 10 y n (%)	268 (83.2)	127 (89.4)	0.090
≥ 10 y n (%)	54 (16.8)	15 (10.6)	
Gender,			
Male n (%)	195 (60.6)	92 (64.8)	0.408
Female n (%)	127 (39.4)	50 (35.2)	
Risk group,			
SR n (%)	117 (36.3)	65 (45.8)	0.063
IR n (%)	205 (63.7)	77 (54.2)	
WBC,			
< 50 × 109 n (%)	267 (82.9)	124 (87.3)	0.269
≥ 50 × 109 n (%)	55 (17.1)	18 (12.7)	
Immunophenotype			
B-ALL n (%)	295 (91.6)	135 (95.1)	0.246
T-ALL n (%)	27 (8.4)	7 (4.9)	
5-year EFS, %	89.1±1.8	87.6±2.8	0.315

WBC: white blood cell; SR: standard risk; IR: Intermediate-risk.

Supplementary Table 2: Genetic polymorphisms and minor allele frequencies

SNP (rs#)	Genes	Alleles (1/2)*	Position*	Function*	MAF
rs11045879	*SLCO1B1*	T/C	chr12: 21229685	Intron	38.7
rs4149056	*SLCO1B1*	T/C	chr12: 21178615	missense, Val>Ala	11.1
rs2306283	*SLCO1B1*	T/C	chr12:21176804	missense, Asn>Asp	74.0
rs10841753	*SLCO1B1*	T/C	chr12:21168436	Intron	30.0
rs1051266	*SLC19A1*	G/A	chr21:45537880	missense, His>Arg	46.5
rs3788200	*SLC19A1*	G/A	chr21:45536657	Intron	46.2
rs1131596	*SLC19A1*	C/T	chr21:45538002	coding synonym, Ser>Ser	54.7
rs2838958	*SLC19A1*	G/A	chr21:45528653	Intron	51.7
rs2032582**	*ABCB1*	G/T	chr7:87531302	missense, Ser>Thr/Ala	48.2
rs1128503	*ABCB1*	T/C	chr7:87550285	coding synonym, Gly>Gly	31.4
rs1045642	*ABCB1*	T/C	chr7:87509329	coding synonym, Ile>Ile	60.8
rs2231137	*ABCG2*	G/A	Chr4:88139962	missense, Val>Met	29.2

*Position and function according to NCBI GRCh38; 1: major allele; 2: minor allele. ** Allele frequency deviates from the Hardy-Weinberg equilibrium. MAF, minor allele frequency.

Supplementary Table 3: Association of haplotypes with plasma levels of MTX at 48 h from the first MTX infusion

Haplotype			Estimated frequency (%)	Median (Min-Max) (µmol/L)	*P*
SLCO1B1 rs10841753	*SLCO1B1* rs2306283	*SLCO1B1* rs4149056			
T	C	T	33.2		
with				0.47 (0.09–41.63)	0.051
without				0.34 (0.12–8019)	
C	C	T	30.1		
with				0.41 (0.11–41.63)	0.524
without				0.50 (0.09–34.05)	
T	T	T	25.6		
with				0.46 (0.12–34.05)	0.933
without				0.44 (0.09–41.63)	
T	C	C	10.8		
with				0.5250 (0.12–17.60)	0.388
without				0.41 (0.09–41.63)	
SLC19A1 rs3788200	*SLC19A1* rs1051266	*SLC19A1* rs1131596			
G	G	T	53.7		0.467
with				0.45(0.09–41.63)	
without				0.48 (0.11–34.05)	
A	A	C	45.5		0.356
with				0.44 (0.09–34.05)	
without				0.60 (0.14–41.63)	

Supplementary Table 4: Association of haplotypes with oral mucositis (grade 3–4)

Haplotype	Chi Square	*P*
SLCO1B1 rs10841753, rs2306283, rs4149056		
TCT	0.998	0.318
CCT	0.355	0.551
TTT	0.508	0.476
TCC	1.723	0.189
SLC19A1 rs3788200, rs1051266, rs1131596		
GGT	0.073	0.786
AAC	0.015	0.902
